# Abnormal signal pathways and tumor heterogeneity in osteosarcoma

**DOI:** 10.1186/s12967-023-03961-7

**Published:** 2023-02-09

**Authors:** Yifeng Sun, Chunming Zhang, Qiongxuan Fang, Wenqiang Zhang, Wei Liu

**Affiliations:** 1 Department of Orthopedic Surgery, The First Affiliated Hospital of Shandong First Medical University & Shandong Provincial Qianfoshan Hospital, Shandong Key Laboratory of Rheumatic Disease and Translational Medicine, Jinan, 250014 Shandong People’s Republic of China; 2 Department of Surgery, Ulm University Hospital, Ulm University, Ulm, Germany; 3 MOE Key Laboratory of Cell Proliferation and Differentiation, School of Life Sciences, Peking University, Beijing, 100871 China

**Keywords:** Osteosarcoma, Abnormal signal pathway, Chemotherapy, Oncocyte heterogeneity

## Abstract

**Background:**

Osteosarcoma (OS) is the most frequent and aggressive primary malignant sarcoma among adolescents and chemotherapy has not substantially progressed for decades. New insights into OS development and therapeutic strategies are urgently needed.

**Methods:**

We analyzed integrated single-cell transcriptomes, bulk RNA-seq, and microarray data from Gene Expression Omnibus (GEO) datasets. We also used Weighted Gene Co-expression Network Analysis (WGCNA), Gene set enrichment analysis (GSEA), and Gene set variation analysis (GSVA), along with Simple ClinVar and Enrichr web servers.

**Results:**

The findings of integrated single-cell analysis showed that OS arises from imperfect osteogenesis during development. Novel abnormalities comprised deficient TGFβ and P53 signal pathways, and cell cycle pathway activation, and a potentially new driver mutation in the interferon induced transmembrane protein 5 (*IFITM5*) that might function as a pathogenic factor in OS. Osteosarcoma is characterized by oncocyte heterogeneity, especially in immunogenic and adipocyte-like subtypes that respectively promote and hamper OS treatment. Etoposide is a promising chemotherapeutic that provides palliation by affecting the subtype of OS and correcting the abnormal pathways.

**Conclusion:**

Various abnormal signal pathways play indispensable roles in OS development. We explored the heterogeneity and underlying mechanisms of OS and generated findings that will assist with OS assessment and selecting optimal therapies.

**Supplementary Information:**

The online version contains supplementary material available at 10.1186/s12967-023-03961-7.

## Introduction

Osteosarcoma (OS) is an extremely aggressive sarcoma for which chemotherapeutic strategies have not substantially progressed for decades [1, 2]. The mechanism of OS awaits explanation and new molecular targets are urgently needed to improve the prognosis of patients. The causes of OS remain unclear in terms of oncogenetic mechanisms, but it might be due to genetic abnormalities, changes in tumor suppressor pathways (such as p53 and Rb), telomerase, and alternative telomere lengthening (ALT) inside bone cells [3]. Osteosarcoma driver mutations have been found in *TP53, RB, PTCH1, MYC, NOTCH1, BRCA2, APC*, and *PRKAR1A* genes. Clinical evaluations of targeted agents have yielded disappointing results [4, 5]. However, some new driver genes are currently under development as potential therapies for OS.

Interrelated biological signaling pathways in OS comprise WNT/βcatenin, Hedgehog, mTOR, and RANKL/NF-κB [6]. Achievements in the underlying molecular biology of OS have contributed to transformative advances in understanding this type of malignancy. Osteosarcoma originates from bone mesenchymal stem cells (MSCs) with osteoblastic lineage commitment. However, understanding the molecular mechanisms of OS development remains insufficient [3, 7]. For example, the TGFβ signal pathway exerts dual effects in terms of tumor prevention and carcinogenesis, depending on the timing of tumor development [8–10].

Osteosarcoma is significantly heterogeneous at the genomic, transcriptomic, and epigenetic levels. Malignant OS cells have stemness properties that are closely involved in chemotherapy resistance, relapse, or refractory and metastatic processes [11–13]. Current approaches to intra-tumor heterogeneity mainly depend on cellular genomic or transcriptional sequencing. However, this lacks high resolution in authenticating the complex cellular subtypes and intra-tumoral heterogeneity in OS. Single-cell RNA-sequencing (scRNA-seq) is a promising tool that can analyze the composition of heterogeneous cell populations and lineage hierarchies [14, 15].

Here we mapped single-cell (sc) RNA sequences of human embryonic long bones, normal bone tissue, and OS at single-cell high resolution to address the development of OS and oncocyte heterogeneity. The results of our integrated bioinformatic analysis identified and validated the process of osteogenesis imperfecta, potential new driver genes, abnormal signaling pathways, transcriptomic heterogeneity, and targeted therapeutic agents for OS.

## Methods

### Single cell RNA sequencing (scRNA-seq) data processing

We analyzed scRNA-seq data using R statistical software (v. 4.1.2, The R Foundation for Statistical Computing, Vienna, Austria), the Seurat v4.0 toolkit and MPLAB Harmony v1.0. Public scRNA-seq data were acquired from the GSE143753, GSE196678, and GSE162454 datasets in the Gene Expression Omnibus (GEO). Low-quality cells (< 3; features, < 200; > 10% mitochondrial genes) were filtered, the data were initially normalized, then batch effects were removed using LogNormalize (features = 3000) and the Harmony function (max.iter.harmony = 20). Dimensions were reduced using Uniform Manifold Approximation and Projection (UMAP). We found marker genes for cell groups in datasets using the COSine similarity-based marker *g*ene identification (COSG) package [16]. We quantified marker genes using Z-scores and the results are shown in a heatmap. We calculated copy number variations to identify malignant oncocytes using the inferCNV package.

Cell types were identified in each subpopulation based on their known lineage markers: mesenchymal cells, vimentin (VIM); immune cells, cluster of differentiation (CD)45; myeloid cells: CD83, CD14, CD68; lymphoid cells, CD3 and B-cell maturation antigen (BCMA); endothelial cells, von Willebrand factor, (VWF) and carcinoembryonic antigen-related cell adhesion molecule-1 (CEACAM1); myogenic cells, myogenic differentiation 1 (MYOD1) and myogenin (MYOG); bone-related cells, collagen type 1 alpha 1 (COL1A1) and alpha 2 (COL1A2) chains, lumican (LUM), SRY-Box Transcription Factor 9 (SOX9), alkaline phosphatase (ALP) and RUNX Family Transcription Factor 2 (RUNX2)).

### Pseudotime and trajectory analysis

Single-cell pseudotime and trajectory analyses were constructed using the Monocle3 toolkit (v. 2.14.0). Evolutionary processes were organized into potentially discontinuous trajectories by the learn_graph function. Pseudotime was defined using the order_cells function with a selected node representing development. Genes that were differentially expressed over these trajectories were then identified [17, 18].

### Weighted gene co-expression network analysis (WGCNA)

We also searched related public expression profiles in 12 MSCs, 3 osteoblasts, and 84 OS samples (GSE33383). A co-expression network was constructed to identify co-expressed modules using the WGCNA package in R. The expression matrix was restricted to only the top 25% of expressed genes according to variance analyses. Relationships between gene sets (modules) were explored using a hierarchical cluster dendrogram. A clustering tree based on the eigengenes of modules calculated the dissimilarity of the module eigengenes (MEs). Associations between gene sets (modules) and clinical features were assessed using Pearson correlations.

### Clinical datasets and resources

We downloaded 53 samples including expression profiles and clinical outcomes of patients with OS from GSE19743 for further analysis. Except for the expression profile, the clinical information mainly included living status, survival duration, Huvos grading scores, and metastatic status. Patients were assigned to high- (n = 27) and low- (n = 26) risk groups based on the median numbers of samples. We applied Cox regression and Kaplan–Meier curves to estimate overall survival.

### Microarray data processing

We used the gene set with the expression profile GSE84500 to obtain significantly differentially expressed genes (DEGs). A data matrix was downloaded to annotate the probe into gene symbol sets. Significance was analyzed using Limma, or the Deseq2 package. The most significant changes in gene expression were identified using false discovery rates (FDR < 0.01) and absolute fold change (FC > 1).

### Gene set enrichment analysis (GSEA)

We assessed gene set enrichment using GSEA [19]. Kyoto Encyclopedia of Genes and Genomes (KEGG) signaling pathway gene sets were curated from the Molecular Signatures Database (Human MSigDB database v2022.1.Hs). Immunogenic and adipocyte-like subtype molecular signatures were created by identifying significant marker genes in scRNA-seq. Normalized enrichment scores (NES) > 1 and p < 0.05 reliably filtered significant pathways.

### Gene set variation analysis (GSVA)

We established the molecular signatures of OS oncocyte subtypes depending on the significant marker genes, which were acquired by single cell analysis [20]. We also calculated final scores for further analyses using GSVA (v. 1.47.0).

### Databases and datasets

Simple ClinVar (https://simple-clinvar.broadinstitute.org/) is a web-server application that summarize variant, gene, gene- and disease-wise statistics based on the entire ClinVar database in a dynamic and user-friendly web-interface [21]. Enrichr (https://maayanlab.cloud/Enrichr/) is a robust web-server that contains many types datasets, among which we used the MGI Mammalian Phenotype, Jensen DISEASES, and BioPlanet pathway databases, as well as the PanglaoDB Augmented, and DrugMatrix datasets [22, 23]. Statistical significance was set at < 0.05.

## Results

### Osteosarcoma arises from osteogenesis imperfecta during development

**Fig. 1:**
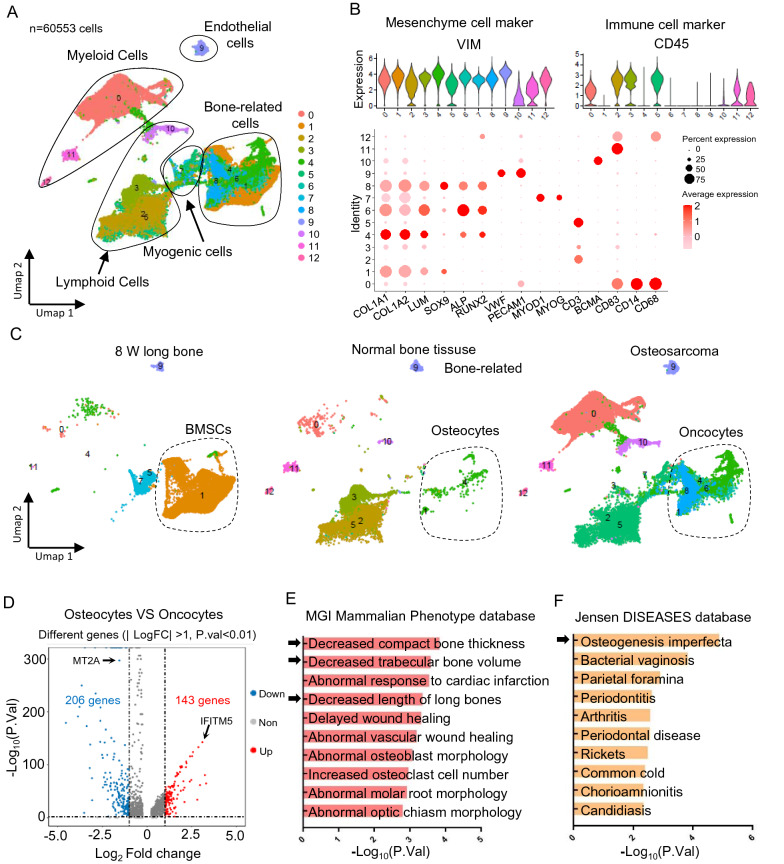
Osteosarcoma arises from osteogenesis imperfecta during development. **A** Single cell RNA-sequence data (scRNA-seq) analysis for 8-week-old long bones (GSE143753), human normal bone tissue (GSE196678) and OS (GSE162454). Cluster analyzed by using Uniform Manifold Approximation and Projection (UMAP) method and populations were identified by color. **B** Markers used to define subpopulations. **C** Separated UMAP depending on tissue origins. **D** Volcano plot of significant different genes (| log2FC | > 1; P < 0.01) between normal osteocytes and OS oncocytes. **E**, **F** Enrichment of MGI Mammalian Phenotype and Jensen DISEASES by using the upregulated genes in **D** (p.value<0.01)

**Fig. 2:**
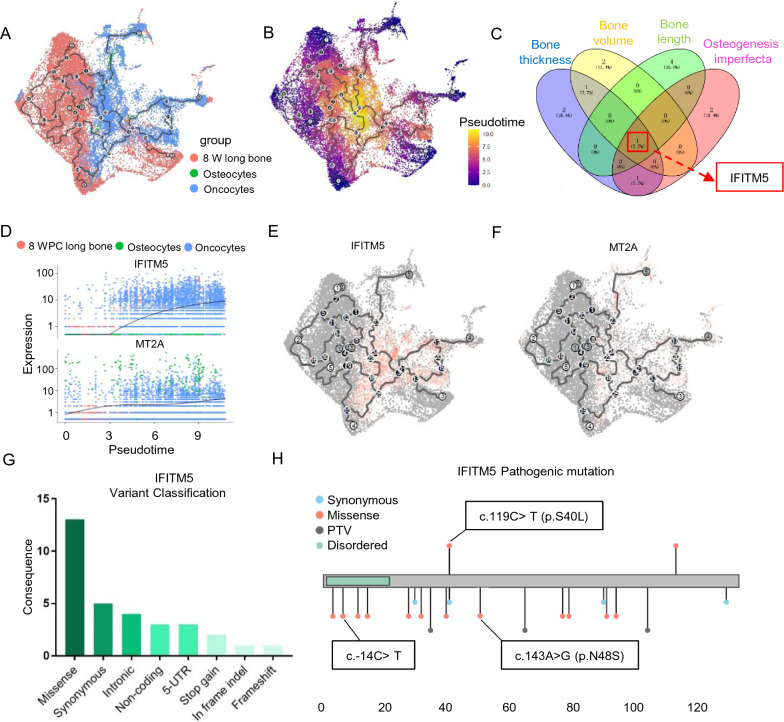
IFITM5 is a potential new driver gene in OS development. **A**, **B** Single-cell trajectory analysis using Monocle3, cells colored by tissue origins and pseudotime. **C** Venn plot show the shared genes among the associated genes in osteogenesis imperfecta, and decreased bone thickness, volume, and length. **D** Plots of expression for ITIFM and MT2A by pseudotime. **E**, **F** UMAP of cell data set branch subsets. Cells are colored by the expression of IFITM5 and MT2A. **G** Various mutation categories of IFITM5 in Simple ClinVar datasets. **H** Coding variant mapping and domain annotation for IFITM5

We integrated data from 8-week-old long bones (GSE143753), human normal bone tissue (GSE196678) and OS (GSE162454) to determine whether a developmental disorder is associated with OS. After quality control and batch correction, we obtained 60,553 cells for downstream analysis. After dimensionality reduction, UMAP-based cell clustering identified myeloid, lymphoid, endothelial, myogenic, and bone-related cell clusters (Fig. 1A). Based on the lineage markers (Fig. 1B), then split them depending on their tissue origins. We found that bone mesenchymal stem cells (BMCs), normal osteocytes, and OS oncocytes mapped together, indicating that oncocytes might be derived from BMSCs due to a developmental disorder (Fig. 1C). We compared transcriptional profiles to identify DEGs between normal osteocytes and OS oncocytes during development. We identified 349 DEGs (log_2_FC > 1; P < 0.01), of which 143 were elevated in OS oncocytes (Fig. 1D; Additional file 2: Table S1). We analyzed the upregulated DEGs using the MGI Mammalian Phenotype and Jensen DISEASES databases to gain insight into their functional relevance. We found enrichment of decreased compact bone thickness, decreased trabecular bone volume, decreased length of long bones, and osteogenesis imperfecta (Fig. 1E and F; black arrows). This further supported the notion of a developmental disorder in OS. We analyzed trajectories to gain insight into the origin and development of bone-related cells (Fig. 2A and B). The results revealed partial BMSCs at the start of the developmental trajectory. This indicated that BMSCs can develop into OS.

### Interferon induced transmembrane protein 5 (*IFITM5*) is a potential new driver gene in OS development

Among the functional ontologies, only *IFITM5* played a crucial role in the process of OS oncogenesis, including osteogenesis imperfecta, and decreased bone thickness, volume, and length (Fig. 2C). Furthermore, *IFITM5* and Metallothionein 2A (*MT2A*) were the most significantly upregulated and downregulated genes, respectively, among the DEGs between normal osteocytes and OS oncocytes (Fig. 1D). The trajectory analyses also offered a clearer view of gene dynamics along a single path. We found that *IFITM5* significantly increased from BMSCs to OS, whereas *MT2A* did not (Fig. 2D). The pseudotime-dependent *IFITM5* gene changed as cells progressed along the oncocyte trajectory. Therefore knowing the order in which *IFITM5* changes can lead to the generation of new models of OS development (Fig. 2E and F). We confirmed the *IFITM5* variant using Simple ClinVar. We summarized various mutation categories of *IFITM5* and found that a missense mutation was the most prevalent type (Fig. 2G), and that three variations of ITITM5, including the point mutation (c.-14C > T), the missense c.119C > T (p.S40L), and c.143A > G (p.N48S), can lead to pathogenic states according to the Simple ClinVar dataset and current literature (Fig. 2H). These findings together indicated that IFITM is a potential new driver gene in OS development that warrants further investigation.

### Osteosarcoma was attributed to complicated regulation of abnormal signal pathways during development

**Fig. 3:**
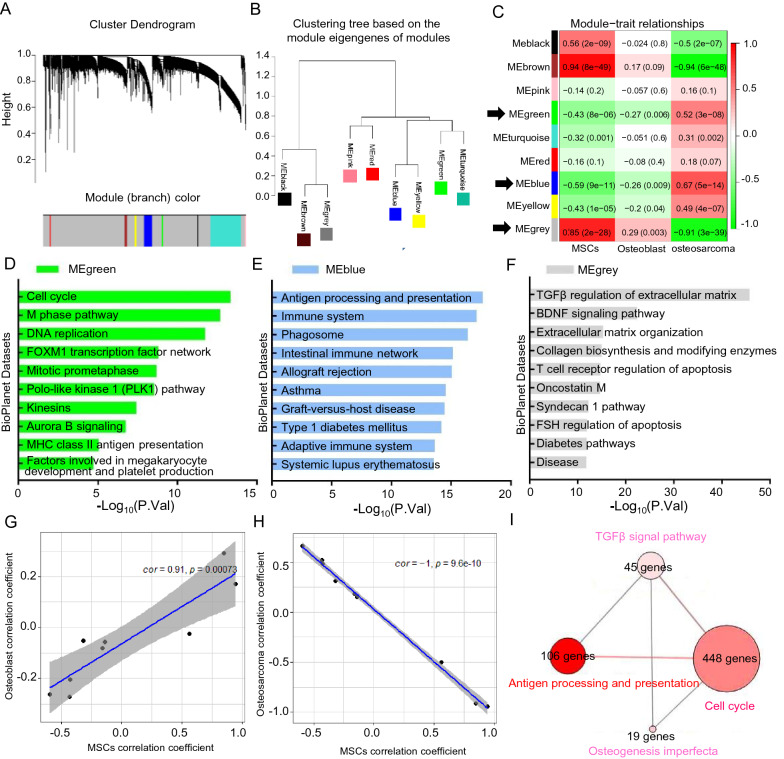
Osteosarcoma was attributed to complicated regulation of abnormal signal pathways during development. **A** Hierarchical cluster dendrogram of identified co-expressed genes in modules in 12 MSCs, 3 osteoblasts, and 84 OS samples (GSE33383). **B** Clustering tree based on the module eigengenes of modules. **C** Heatmap of the correlation between hub genes and cell types. **D**, **E**, **F** Enrichment of BioPlanet signal pathway in MEgreen, MEblue, and MEgrey modules. **G**, **H** The correlation analysis between MSCs and osteoblast, as well as MSCs and osteoblast by Pearson method. **I** The connections of atypical signal pathways in ConsensusPathDB datasets

We further investigated OS development by analyzing the expression profiles of 12 MSCs, 3 osteoblasts, and 84 OS samples (GSE33383). We established correlation networks and identified co-expression modules using WGCNA. Nine co-expression modules with 4,637 genes were obtained using the Dynamic Tree Cut algorithm (Fig. 3A). The clustering tree mainly showed two branches based on the module eigengenes (Fig. 3B). Correlations between modules and samples of MSCs, osteoblasts, and OS are shown in (Fig. 3C). The heat map shows that MEgreen and MEblue modules correlated negatively with MSCs and osteoblasts, and positively with OS. The MEgery module correlated positively with MSCs and osteoblasts, but negatively with OS. We analyzed BioPlanet pathways using the co-expressed genes in the MEgreen, MEblue and MEgery modules to gain insight into the signal pathway relevance (Additional file 3: Table S2). The three most significant pathways in each module, namely, Cell cycle, Antigen processing and presentation, and TGFβ regulation of extracellular matrix were enriched (Fig. 3D‒F). The module correlation coefficients for MSCs were consistent with those of osteoblasts, in contrast to OS (Fig. 3G and H). That is, OS was attributed to constitutive activation of the Cell cycle and Antigen processing and presentation pathways, and deficient TGFβ regulation of extracellular matrix signals. The atypical signal pathways also had complex connections to regulate bone development (ConsensusPathDB; Fig. 3I).

### Transcriptomic heterogeneity of oncocytes in OS

**Fig. 4:**
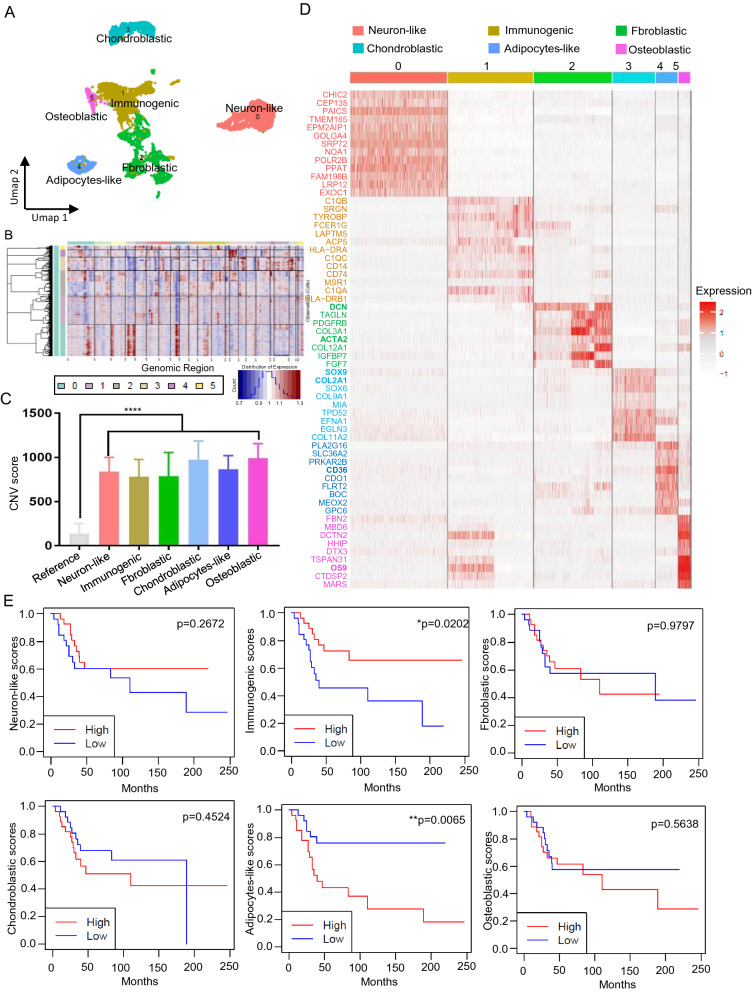
Transcriptomic heterogeneity of oncocytes in OS. **A** Re-clustering of OS oncocytes. **B** The copy number variation (CNV) was estimated using inferCNV (with myogenic cells as a reference) to distinguish oncocytes and normal cells. The red and blue colors represent the copy number variation. **C** The CNV score of each cell was calculated as a quadraic sum of CNV. **D** A heat map shows the hub genes in each sub-cluster. **E** Kaplan–Meier survival curve shows the survival of OS patients with high- and low risk-scores for each subcluster signature (Nhigh-risk = 27 vs. Nlow-risk = 26)

We analyzed the transcriptomic heterogeneity of OS oncocytes by re-clustering bone-related cells in OS (n = 8758 cells) and found six distinct transcriptomic sub-clusters (Fig. 4A). The positive expression of COL1A1, COL1A2, ALP and the absence of CD45 revealed that the six sub-clusters were bone-related oncocytes (Additional file 1: Fig. S1 B). The copy number variation (CNV) was estimated using inferCNV (with myogenic cells as a reference) to distinguish oncocytes and normal cells (Fig. 4B). The CNV from scRNA-seq revealed numerous alterations in all sub-clusters compared with the reference cells (Fig. 4C). We determined the top 100 differentially expressed transcripts among the six sub-populations and defined their cell types in the Enrichr datasets (Additional file 4: Table S3). A heat map shows the hub genes in each sub-cluster (Fig. 4D). Six sub-populations were annotated and visualized by UMAP as neuron-like, immunogenic, fibroblastic, chondroblastic, adipocyte-like, and osteoblastic oncocytes (Fig. 4A). The top 100 gene signatures of each sub-population were assessed using GSVA and the oncocyte subtype abundance was estimated from 53 human transcriptomic profiles (GSE21257). Kaplan–Meier estimates associated higher immunogenic and adipocyte-like scores with longer progression-free survival (p = 0.0202), whereas and unfavorable prognoses, respectively (p = 0.0065). Other subtypes had no significant prognostic value (Fig. 4E). IFITM5 is a potential new driver gene in OS development in our study. Correlation analysis was performed to further identify the relationship between tumor subtypes and IFITM5. We found that the expression of IFITM5 has a strongly significant positive correlation with adipocyte-like and chondroblastic subtypes and a significant negative correlation with immunogenic and fibroblastic subtypes. Therefore, the high expression of IFITM5 was associated with the shorter overall survival subtype which is in line with the previous study (Additional file 1: Fig. S1A) [24].

### Deficient TGFβ signaling leads to adipocyte-like subtype of OS during development

**Fig. 5:**
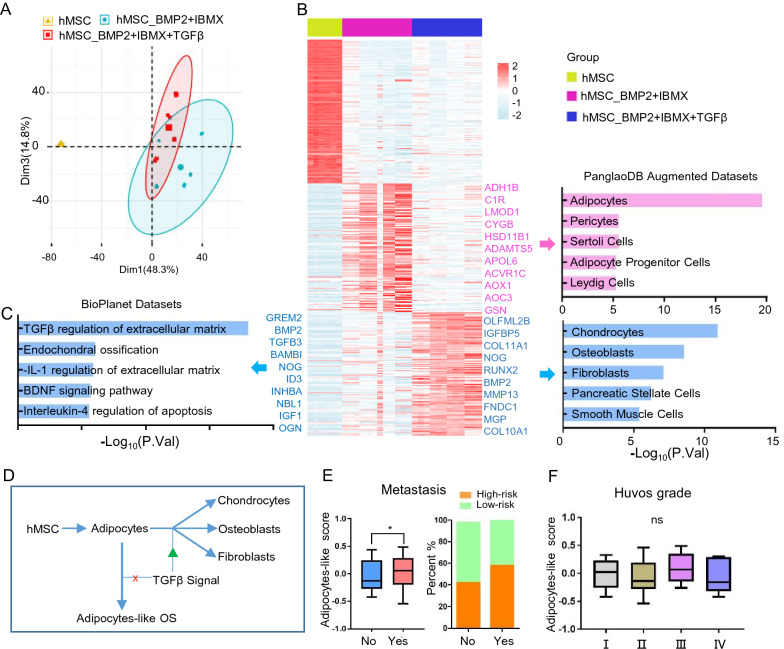
Deficient TGFβ signaling leads to adipocyte-like subtype of OS during development. **A** Principal component analysis (PCA) biplot of transcriptional profiles with 95% confidence ellipsoids in the panels of hMSC, hMSC_BMP2+IBMX and hMSC_BMP2+IBMX+TGFβ (GSE84500). **B** Heat map shows the significantly different genes in these three groups (log2FC > 1; P < 0.01), and enrichment of cell types in PanglaoDB Augmented Datasets. **C** Enrichment of BioPlanet signal pathway in hMSC_BMP2+IBMX+TGFβ group. **D** Sketch map of TGFβ signaling in OS development. **E** Box plot and corresponding stacked histogram demonstrate the correlation between metastasis and adipocyte-like subtype score. **F** Huvos grading system was used to judge the effectiveness of neoadjuvant chemotherapy on OS

The adipocyte-like OS subtype was associated with a poor prognosis. We profiled gene expression in human MSCs (hMSCs) in the presence of adipogenic/osteogenic factors (GSE84500) to assess the development of adipocyte-like subtype of OS. A comparison of transcriptional profiles between panels of hMSC_BMP2 + IBMX and hMSC_BMP2 + IBMX + TGFβ. Principal Component Analysis (PCA) initially revealed correlations (Fig. 5A). We identified 469 upregulated transcripts (log2FC > 1, P < 0.01), of which 240 were elevated in the hMSC_BMP2 + IBMX group (Additional file 5: Table S4) and named Adipocytes in PanglaoDB Augmented Datasets and 229 that were abundantly expressed in the hMSC_BMP2 + IBMX + TGFβ group (Additional file 6: Table S5) were labeled Chondrocytes, Osteoblasts, and Fibroblasts in the same datasets (Fig. 5B). Meanwhile, BioPlanet pathways confirmed activated TGFβ regulation of extracellular matrix in the hMSC_BMP2 + IBMX + TGFβ group. Therefore, we judged that BMP2 and IBMX cause hMSCs to differentiate into adipocytes, TGFβ inhibits this process and redirects these cells to differentiate into chondrocytes, osteoblasts, and fibroblasts. The TGFβ signal was defective during OS development (Fig. 3C and F), and adipocytes could not differentiate into normal bone cells without the TGFβ signal. This stalled adipocyte development and led to the adipocyte-like subtype of OS (Fig. 5D). In clinical translation, patients with metastasis are more likely to have high-risk scores (Fig. 5E). We applied the Huvos grading system to judge the effectiveness of neoadjuvant chemotherapy on OS; higher scores reflect larger necrotic areas in the clinical data (GSE21257). However, the risk score of the adipocyte-like subtype did not differ among grades, indicating that the adipocyte-like subtype is resistant to neoadjuvant chemotherapy (Fig. 5F).

### Etoposide exerts effective palliation by affecting the subtype of OS and correcting abnormal pathways

**Fig. 6:**
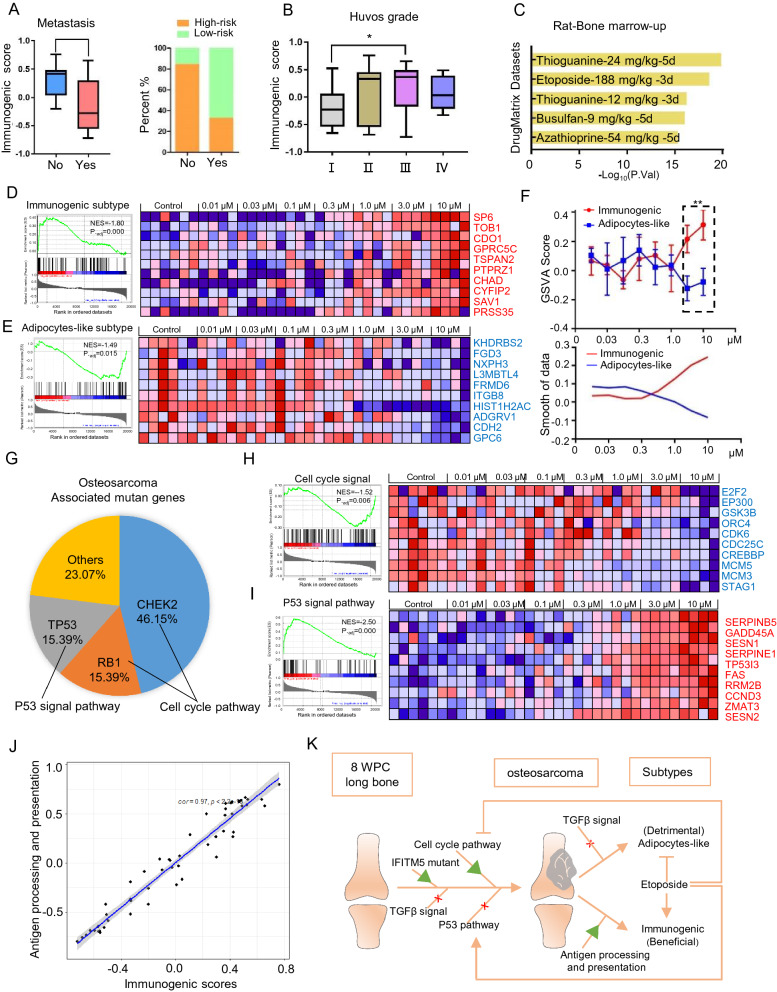
Etoposide exerts effective palliation by affecting the subtype of OS and correcting abnormal pathways. **A** Box plot and corresponding stacked histogram show immunogenic subtype score in metastasis and no metastasis OS samples. **B** Immunogenic subtype score in OS samples across various Huvos grades. **C** Enrichment of immunogenic subtype gene signature in DrugMatrix dataset. **D**, **E** The GSEA enrichment plot and dose-dependent heatmap showed the effect of etoposide on immunogenic and adipocyte-like subtypes. **F** GSVA analysis was used to determine the different tendencies between immunogenic and adipocyte-like subtypes with different concentrations of etoposide. **G** Osteosarcoma associated mutant genes in Simple ClinVar datasets. **H**, **I** The GSEA enrichment plot and dose-dependent heatmap revealed the effect of etoposide on the cell cycle and p53 signaling pathway. **J** The correlation analysis between immunogenic subtype and Antigen processing and presentation pathway. **K** Overview of the abnormal signal pathways and tumor heterogeneity in osteosarcoma

Patients with OS and higher-, than lower-risk scores for the immunogenic subtype survived longer (Fig. 4E) and metastasis was less likely to develop (Fig. 6A). Furthermore, the Huvos system associated the immunogenic subtype with higher grades and sensitivity to neoadjuvant chemotherapy (Fig. 6B). We applied the immunogenic subtype gene signature to the DrugMatrix dataset that contains comprehensive information about gene expression in rats under treatment with various drugs. Transcriptional changes caused by thioguanine and etoposide were enriched (Fig. 6C). An extensive literature review revealed that etoposide is a feasible candidate as salvage therapy for relapsed and metastatic OS. A high-throughput transcriptomic screen of OS cells (U-2 OS) exposed to seven concentrations of etoposide was evaluated by GSEA (GSE200845) to determine the underlying mechanism of etoposide. The GSEA enrichment plot associated DEGs with the activation of immunogenic subtype signatures (p = 0.000, NES = 1,80; Fig. 6D left). The heat map also shows that the top 10 genes with immunogenic subtype signatures gradually increased as the dose increased (Fig. 6D right). Meanwhile, the enrichment plot and a heat map showed that the adipocyte-like subtype signatures correlated negatively with the dose-dependent profile of etoposide treatment (p = 0.015; NES = − 1.49; Fig. 6E). We also used GSVA to determine a dose-dependent tendency between immunogenic and adipocyte-like subtype signatures. The results were the same as those of the GSEA analysis (p < 0.01, Fig. 6F). We identified the high-risk genes checkpoint kinase 2 (CHEK2) and retinoblastoma transcriptional corepressor 1 (RB1), which represented 46.15% and 15.39% of the OS driver genes respectively, and play crucial roles in the cell cycle pathway, which we confirmed was activated (Figs. 6G; 3D). The mutation rate of the important tumor suppressor gene tumor protein 53 (TP53) was also 15.39% in OS (Fig. 6G). Etoposide can inhibit the cell cycle pathway, and activate the P53 signaling pathway (Fig. 6H and I). The immunogenic OS subtype originated in MSCs, because it correlated closely with Antigen processing and presentation as described in Fig. 3E (Fig. 6J). Therefore, etoposide has promise as a palliative therapeutic by promoting immunogenic subtypes, inhibiting adipocyte-like subtypes, and correcting the abnormal cell cycle and p53 signaling pathways.

## Discussion

Osteosarcoma is the most frequent primary malignant sarcoma in adolescents. Given the treatment dilemma, new insights into the OS development and therapeutic strategies are urgently needed [25, 26]. Here, we discovered using integrated single-cell analysis, that OS arises from osteogenesis imperfecta during development. We identified deficient TGFβ and P53 signal pathways, an activated cell cycle pathway, and a potentially novel driver gene *IFITM5* mutant as potential pathogenic factors of OS. We characterized OS heterogeneity and found that the immunogenic and adipocyte-like subtypes respectively exerted beneficial and detrimental effects on OS. Etoposide is a promising chemotherapeutic drug that achieves palliation by affecting the OS subtypes and correcting aberrant pathways.

Osteosarcoma is derived from MSCs and is characterized by osteogenesis imperfecta [27, 28]. The *IFITM5* gene encodes an osteoblast-specific membrane protein that is an established positive regulatory factor during bone mineralization [29, 30]. Single base variants (c.119 C > T), c.143A > G and (c.-14 C > T) in the coding region of *IFITM5* have been identified in patients with osteogenesis imperfecta type V [31, 32]. Furthermore, *IFITM5* is overexpressed in abnormal bone hyperplasia in rat primary osteoblasts, UMR106 rat osteosarcoma cells, human primary osteoblasts and Saos-2 human osteosarcoma cells [33–35]. There are already proven cases published about osteosarcoma occurring in osteogenesis imperfecta due to IFITM5 mutation [36, 37]. The skeletal disorders caused by alterations in the IFITM5 gene, including c.-14C > T, c.119C > T (p.S40L), c.143A > G (p.N48S), while more case series are required to establish detailed genotype-phenotypes for these alterations in the IFITM5 gene [32, 38]. Here, we determined that IFITM is a potential new driver gene in OS development depending on pseudotime using trajectory analysis and the ClinVar database.

The various abnormal signal pathways that prompt MSCs towards abnormal bone growth also play indispensable roles in OS development [6, 25]. The dysregulated cell cycle (CHEK2, RB1 mutant) and p53 (TP53 deficiency) signaling pathways often result in pathogenesis and aberrant OS growth. The p53 signal pathway acts as a tumor suppressor and exerts a crucial role in safeguarding our body from developing OS [39]. There are many studies reported that human osteosarcomas can have a deletion, mutations, and/or rearrangements of the p53 gene, which may cause loss of normal constraints on cellular growth, cell cycle, senescence and metabolism [40–42].P53 also regulates osteogenic, chondrogenic, myogenic, adipogenic differentiation of MSCs [43, 44]. The present findings are also in line with the fact that Rb and p53 knockout in bone-marrow-derived MSCs (BM-MSCs) results in OS-like tumors that can be serially transplanted [45, 46]. The TGF-β signal favors normal bone formation in the mesenchymal osteoblastic lineage [47, 48]. However, TGF-β seems to mainly have a pro-tumoral effect on OS [49–52]. Several clinical strategies to block TGF-β signaling pathways, such as neutralizing antibodies (GC-1008), ligands or receptors inhibitors (AP12009), or chemical compounds (SB-431542, or LY2157299), have not been successful against OS [10, 53–56]. In contrast to previous findings, we have the new perspective that a TGF-β deficiency is a major cause of OS and leads to a detrimental adipocyte-like subtype. This is an alternative experimental approach to treating patients with OS. Briefly, the TGF-β signal pathway in MSCs guards bone formation in the early stage and then compromises their osteogenic differentiation in the late stage, therefore it dictates the conditions for bone development [47, 57]. In this context, TGF-β signal pathway mutations and/or alterations mutations to TGF-β cascade components have been associated with several bone disorders and many carcinomas [58, 59].Several high-throughput genomic and transcriptomic studies have delineated the intra-tumoral heterogeneity of OS [11, 60–62]. Zhang et.al identified seven OS tumor cell clusters with three differentiation branches by using single-cell RNA sequencing of conventional OS and cancellous bone (CB) samples, which have different prognoses and possible drug sensitivities [63]. Jiang et.al classified OS into four subtypes according to the genomic, epigenomic, and transcriptomic data, while our classification depended on the single cell sequencing. We used different methods to identify the heterogeneity of OS, but partially with similar molecular features and clinical prognosis, such as Immune activated (S-IA) and our immunogenic subtype both have immunological traits and better clinical prognosis. Their Immune suppressed (S-IS) subtype has activated adipogenesis and fatty acid metabolism-related pathways and encodes the fatty acid scavenger receptor CD36 which is in line with our adipocyte-like subtype [64]. We explored the heterogeneity and the underlying mechanism of OS that could help to provide better, customized therapy. We identified six oncocyte subtypes of OS, of which four did not affect prognosis (Additional file 1: Fig. S1C and D). The immunogenic and adipocyte-like subtypes were respectively associated with a better and worse prognosis. To our knowledge, this is a novel molecular signature classification of OS, which will facilitate OS assessment, and choosing the optimal therapy. For example, etoposide is associated with the activation and inhibition, respectively, of the immunogenic and adipocyte-like subtype signatures, and combining it with other strategies has shown promising anti-tumour activity in clinical trials [65–68].

However, it should be noted that the study we examined is limited to bioinformatics analysis, and a significant number of challenges still need to be overcome for a successful in vitro and vivo study. Large-scale samples including both single-cell sequencing and clinical outcomes should be taken into account in the future. In addition, Some different signaling networks in osteosarcoma, including RANKL/RANK, Wnt, Notch, PI3K/Akt/mTOR, and mechanotransduction pathways, contribute to osteosarcoma progression and metastasis and tumor heterogeneity, which certainly looks worthy of further investigation.

## Conclusions

We attributed the occurrence of OS to an *IFITM5* mutant, deficient TGFβ signaling, continuous activation of the cell cycle signal pathway, and P53 signal inhibition during development. Among six OS subtypes, the immunogenic subtype was beneficial, whereas the adipocyte-like subtype was detrimental to health. Etoposide could be a useful palliative because it altered the OS subtype, inhibited the cell cycle pathway, and improved the p53 signal pathway.

## Supplementary Information

**Additional file 1: Fig.S1.** A The correlation analysis between IFITM5 expression and each tumor subtype. B Markers used to define subpopulations. C Box plots show the correlation between metastasis and other subtype scores. D Huvos grades of neuron-like, fibroblastic, chondroblastic, and osteoblastic subtypes.

**Additional file 2: Table S1.** Different genes between normal osteocytes and OS oncocytes.

**Additional file 3: Table S2.** Signatures of each module in WGCNA analysis.

**Additional file 4: Table S3.** Signatures of each OS subtype.

**Additional file 5: Table S4.** Significantly different genes of the hMSC_BMP2+IBMX group.

**Additional file 6: Table S5.** Significantly different genes of the hMSC_BMP2+IBMX+TGFβ group.

## Data Availability

All data generated or analyzed during this study are included in this published article and its supplementary information files.

## Abbreviations

ALP: Alkaline phosphatase
ALT: Alternative telomere lengthening
APC: Antigen presenting cell
BCMA: B-cell maturation antigen (endothelial cells)
BM-MSCs: Bone marrow mesenchymal stem cells
BMP2: Bone morphogenetic protein 2
BRCA2: Breast cancer gene 2
CD: Cluster of differentiation
CEACAM1: Carcinoembryonic antigen-related *cell* adhesion molecule-1
CHEK2: Checkpoint kinase 2
CNV: Copy number variation
COL1A1: Collagen type 1 alpha 1
COL1A2: Collagen type 1 alpha 2
COSG: *COSine similarity-based marker Gene identification*
GEO: Gene Expression Omnibus
GSEA: Gene set enrichment analysis
GSVA: Gene set variation analysis
hMSC: Human mesenchymal stem cells
IBMX: 3-Isobutyl-1-methylxanthine
IFITM5: Interferon induced transmembrane protein 5
LUM: Lumican
MT2A: Metallothionein 2A
mTOR: Mammalian target of rapamycin
MYC: Myelocytomatosis proto-oncogene
MYOD1: Myogenic differentiation 1
MYOG: Myogenin
NF-κB: Nuclear factor kappa B
NOTCH1: Neurogenic locus notch homolog protein 1
OS: Osteosarcoma
PRKAR1A: Protein Kinase CAMP-Dependent Type I Regulatory Subunit Alpha
PTCH1: Protein patched homolog 1
RANKL: Receptor activator of nuclear factor kappa beta
RB1: Retinoblastoma transcriptional corepressor 1
RUNX2: RUNX Family Transcription Factor 2
scRNA-seq: Single-cell RNA-sequencing
SOX9: SRY-Box Transcription Factor 9
TGFβ: Transforming growth factor beta
TP53: Transcriptional repressor 53
UMAP: Uniform Manifold Approximation and Projection
VIM: Vimentin
VWF: Von Willebrand factor
WGCNA: Weighted Gene Co-expression Network Analysis
WNT: Wingless-related integration site
