# Supplementary figures and images for: Abnormal signal pathways and tumor heterogeneity in osteosarcoma
Source: J Transl Med. 2023 Feb 9;21:99. doi: 10.1186/s12967-023-03961-7 (PMC9912612; doi:10.1186/s12967-023-03961-7)

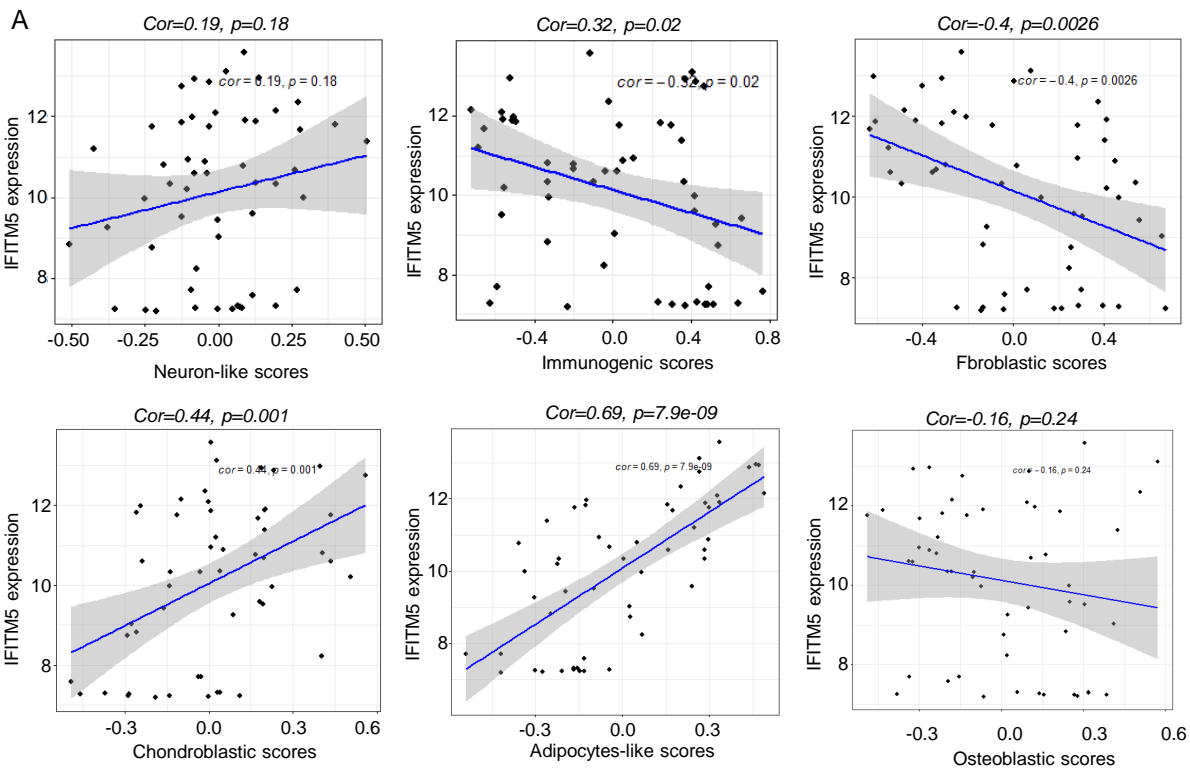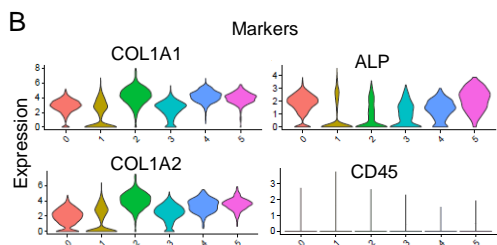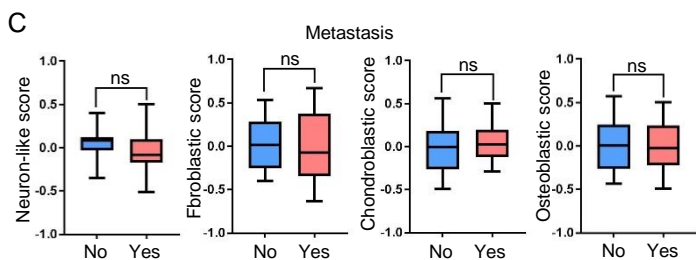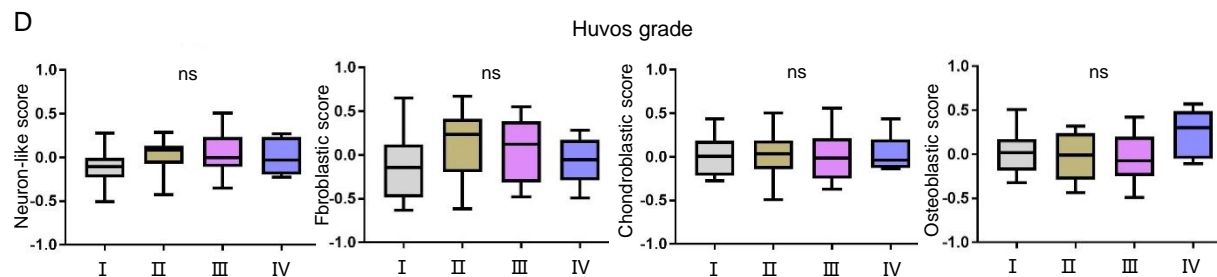

Supplement: Supplementary file 1 — Additional file 1: Fig.S1. A The correlation analysis between IFITM5 expression and each tumor subtype. B Markers used to define subpopulations. C Box plots show the correlation between metastasis and other subtype scores. D Huvos grades of neuron-like, fibroblastic, chondroblastic, and osteoblastic subtypes. [file 12967_2023_3961_MOESM1_ESM.pdf]
